# Health care costs and resource utilization for different asthma severity stages in Colombia: a claims data analysis

**DOI:** 10.1186/s40413-018-0205-4

**Published:** 2018-11-12

**Authors:** Álvaro Flórez-Tanus, Devian Parra, Josefina Zakzuk, Luis Caraballo, Nelson Alvis-Guzmán

**Affiliations:** 1 Health Economics Research Group, University of Cartagena, Campus Piedra de Bolívar, Cartagena, Colombia; 2 Center for Research and Innovation in Health, Coosalud, Street 11 – 2 Floor 8, Bocagrande, Cartagena, Colombia; 3 Institute for Immunological Research, University of Cartagena, Campus de Zaragocilla, Edificio Biblioteca Primer piso, Cartagena, Colombia; 4 Foundation for the Development of Medical and Biological Sciences (Fundemeb), Cra 5 #7-77, Cartagena, Colombia; 5 Hospital Management and Health Policy Research Group, Universidad de la Costa, Barranquilla, Colombia; 6 ALZAK Foundation, Calle 70 #6-99, Cartagena, Colombia

**Keywords:** Cost of illness, Health care costs, Asthma, Health care utilization

## Abstract

**Background:**

Asthma is one of the most common chronic respiratory conditions worldwide. Asthma-related economic burden has been reported in Latin America, but knowledge about its economic impact to the Colombian health care system and the influence of disease severity is lacking. This study estimated direct medical costs and health care resource utilization (HCRU) in patients with asthma according to severity in Colombia.

**Methods:**

This study identified all-age patients who had at least one medical event linked to an asthma diagnosis (CIE-10: J45-J46) between 2004 and 2014. Patients were selected if they had a continuous enrollment and uninterrupted insurance coverage between January 1–2015 and December 31–2015 and were categorized into 4 different severity levels using a modified algorithm based on Leidy criteria. Healthcare utilization and costs were estimated in a 1-year period after the identification period. A Generalized Linear Model (GLM) with gamma distribution and log link was used to analyze costs adjusting for patient demographics.

**Results:**

A total of 20,410 patients were included: 69.5% had mild intermittent, 18.0% mild persistent, 6.9% moderate persistent and 5.5% severe persistent asthma; with mean costs (SD) of $67 (134), $482 (1506), $1061 (1983), $2235 (3426) respectively (*p* < 0.001). The mean total direct cost was estimated at $331 (1278) per patient. Medication and hospitalization had the higher proportion in total costs (46% and 31% respectively). General physician visits was the most used service (57.2%) and short-acting β-2 agonists the most used medication (24%).

**Conclusions:**

Health services utilization and direct costs of asthma were highly related to disease severity. Nationwide health policies aimed at the effective control of asthma are necessary and would play an important role in reducing the associated economic impact.

**Electronic supplementary material:**

The online version of this article (10.1186/s40413-018-0205-4) contains supplementary material, which is available to authorized users.

## Background

As the most prevalent chronic respiratory disease worldwide, asthma contributes enormously to the total economic burden of non-communicable diseases [1–3]. This chronic and difficult-to-treat condition demands high expenditures in medical care services and impairs quality of life and productivity of patients [4, 5]. Recent estimations indicated that asthma caused 1.1% of global disability-adjusted life years (DALYs). From 1990 to 2015, its global prevalence increased by 12.6%, affecting 358.2 million all-age individuals worldwide [6–8].

Annual asthma-related direct costs are highly variable among countries. Estimations have been reported from less than US$150 per person-year in Abu Dhabi, United Arab Emirates to more than US$3000 per person-year in the United States (US) [9]. Disease severity is considered a major factor influencing health care resource utilication (HCRU) and related costs. Even though severe asthma is not common, its contribution to total costs is high [10–13].

Despite some pharmacological advances and the divulgation of guidelines for diagnosis, treatment and prevention, asthma is still a global difficult-to-treat condition and the implementation of strategies for achieving better outcomes remains heterogeneous, especially in developing countries [14–18]. In Latin America, as in other developing regions in the world, several barriers exist to achieve asthma control [19–21]. Disease mechanisms are poorly understood and co-existence of infectious and chronic diseases represents major challenges to health care systems [22–25]. As a result, high rates of uncontrolled and severe asthma have been reported and asthma remains neglected as a public health priority [26–28].

Colombia is a predominantly urban country (76%) of over 48 million inhabitants where chronic diseases are emerging as public health concerns [29]. In 1993, the Colombian Congress sanctioned the Law 100, which replaced the former National Health System (NHS) and introduced a healthcare system known as “Sistema General de Seguridad Social en Salud (SGSSS) (The General System of Social Security in Health)”, an obligatory national health insurance system based on regulated competition. Formally employed individuals, retirees or self-employed individuals earning at least the minimum wage must contribute to SGSSS through a mandatory payroll deduction (contributive regime) and individuals from the low-income population (near 23 million) are affiliated through governmental subsidies (subsidized regime). Currently, almost of 95% of the population is covered by SGSSS and both have equal access to healthcare services.

A nationwide study conducted in Colombia estimated a prevalence rate of 12% (95% CI: 10.5–13.7) for current asthma symptoms suggesting an increase compared to a 10% rate (95% CI: 9.7–11.1) reported 10 years earlier. Furthermore, 43% of subjects with reported asthma symptoms also informed requiring an emergency department (ED) visit or hospitalization in the past year [30, 31]. Nevertheless, little is known about the economic burden of asthma in Colombia and cost of illness studies assessing the influence of disease severity in direct costs and HCRU have not been conducted.

There are no studies in Latin America assessing asthma-related costs through claim-based approaches and micro-costing methodologies in spite of their usefulness as inputs for economic evaluations. Thus, current estimations are associated with uncertainty as high variability in study designs exists. Determining the costs of asthma will help to identify the main expenditure predictors, to analyze if current spending is allocated effectively and to suggest how it should be invested in the future. Assessing the direct medical costs is a valuable input in the design, monitoring and evaluation of health policies and strategies for disease management. In addition, targeting strategies to approach specific groups of the population with asthma according to cost predictors, may contribute to reduce the resulting economic burden and therefore to improve efficiency in the resource allocation process. In this study, we sought to estimate the HCRU and direct medical costs of asthma for different severity stages from the Colombian health care system perspective.

## Methods

### Data source

The primary data source for cost estimation was a claims database from a subsidized regime insurance company of national coverage (with almost two million affiliates) that provides health services to the poorest populations that are not affiliated to the health system through formal employment. Person-level information on demographics, HCRU and total expenditures were available for the study.

### Study design

**Fig. 1:**
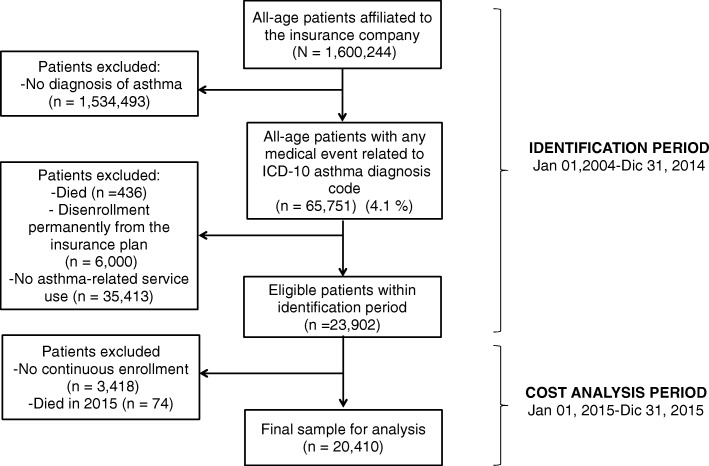
Patient identification and selection process

This was a retrospective analysis of a claims database to estimate the utilization of medical services and asthma-related medication within an open cohort of asthmatic patients. We used a stepwise process to identify and select patients for inclusion as well as for disease severity assessment. Patients were identified from January 1, 2004 through December 31, 2014. An “asthma patient” was defined as any occurrence of an International Classification of Diseases, Tenth Revision (ICD-10) code J45 and/or J46 linked to a medical event such as outpatient visit, ED visit or hospitalization during the identification period. Then, to select the most accurate sample among the eligible patients, an individual must have fulfilled a continuous enrollment and uninterrupted health insurance coverage for at least 12 months during the cost analysis period (from January 1, 2015 through December 31, 2015) (Fig. 1) to be included in the study. In addition, to ensure quality of information, a physician of the research staff revised image/laboratory procedures and prescription data from selected cases to filter and include only those derived from asthma management and not with other unrelated co-morbidity.

### Disease severity classification

Asthma severity classification was mainly based on Leidy criteria [32, 33]. This algorithm has been used previously in other administrative claims studies [34–36] and is based on the number of β2-agonist inhalers and oral corticosteroid (OCS) fills used during the year of cost analysis. The number of exacerbations was also included in the algorithm of classification for minimizing the likelihood of underassessment of severity. An exacerbation was defined as an ED visit or hospitalization within the cost analysis period.

Mild intermittent asthma was defined as one (or less) SABA fill and zero oral OCS fills per year and zero exacerbations. Mild persistent asthma was defined by four to six SABA fills and zero oral OCS fills per year, or two to three SABA fills and less than two OCS fills per year, or one exacerbation. Furthermore, one (or less) SABA fill and one oral OCS fills per year can also account for mild persistent asthma. Moderate persistent asthma included more than six SABA fills and less than two OCS fills per year, or four to six SABA fills and one to two OCS fills per year or 2–3 exacerbations. Patients with severe persistent asthma required to have more than six SABA fills per year, and the number of OCS fills per year, was greater than or equal to two or 4 or more exacerbations. Moreover, zero to six SABA fills and three or more SABA fills per year also constitute severe persistent asthma [33].

### Patient information

Demographic (i.e., age, sex and place of residence) and socioeconomic characteristics (the category in the information system used in Colombia to identify potential beneficiaries of public spending – SISBEN) [37] of the final sample were stratified and compared among asthma severity categories. Associated comorbidities were identified on the claims database as the additional occurrence of their respective ICD-10 codes on the sample of asthmatic subjects.

### Direct costs

Costs derived from medical services: hospitalizations, ED visits, outpatient visits, specialized care and other ambulatory services (i.e., domiciliary care, laboratory/image procedures and ambulances) as well as prescriptions of controller or rescue medications (see Additional file 1) were obtained directly from the billing database of the health insurance company. The cost *per patient* was calculated in each type of health care resource (or medication category) as the sum of the cost of the respective events reported during 2015. Mean costs were estimated among all subjects belonging to each disease category or among only those who used the medical service or received the prescription. The total cost *per patient* including all expended medical services and all prescriptions (or both) was also calculated to further estimate mean total costs. To allow for cost comparability, 2015 purchasing power parity (PPP) exchange rate from the World Bank International Comparison Program Database was used to convert costs estimations to 2015 International Dollars (I$). Total expenditures were divided by the PPP exchange rate ($1203.9 COP) [38]. Purchasing Power Parity adjustment allows a comparable analysis of economic data from different countries by expressing expenditures in a common price index (i.e., based in US) due to potential differences in purchasing powers between countries despite conversion using market exchange rates. A PPP exchange rate is the number of units of a country currency required to purchase the same amounts of goods and services in the domestic market as a US dollar would buy in the US. An International Dollar is therefore a hypothetical currency that has the same purchasing power as the US Dollar has in the US for similar goods and services [39–41].

### Sensitivity analysis

We analyzed the impact of other respiratory co-morbidities and allergic diseases on asthma-related direct costs by performing a sensitivity analysis. All patients with any of the selected co-morbidities (i.e., rhinitis, atopic eczema, acute bronchitis, pulmonary emphysema and COPD) were excluded and total costs compared with the entire database. Sub-group analyses in age-categories with more potential of misclassification with other respiratory diseases were also done.

### Statistical analysis

Data analysis was performed in Stata 14 software (StataCorp LP Lakeway, TX, US) and R statistical software version 3.3.4. Descriptive information about clinical and sociodemographic data of patients involved in the study is shown in Table 1. Differences between proportions were analyzed by Pearson chi-squared test. The Cochran-Armitage test was used for trend association analysis.

**Table 1:** Patient characteristics at baseline in relation to asthma severity - 2015

	All patients *n* = 20,410	Mild intermittent *n* = 14,191	Mild Persistent *n* = 3680	Moderate Persistent *n* = 1408	Severe persistent *n* = 1131	*p*-value ^a^
Characteristic						
Age in years, mean (SD)	23.9 (24.6)	23.4 (23.9)	23.7 (25.0)	27.4 (27.8)	26.3 (27.2)	< 0.001
Age, n (%)						
0-4y	7377 (36.1)	4913 (34.6)	1426 (38.7)	543 (38.5)	495 (43.7)	< 0.001
5-9y	3060 (14.9)	2280 (16.0)	482 (13.1)	178 (12.6)	120 (10.6)	
10-14y	1619 (7.9)	1174 (8.2)	300 (8.1)	93 (6.6)	52 (4.6)	
15-19y	804 (3.9)	614 (4.3)	135 (3.6)	32 (2.2)	23 (2.0)	
20-44y	2964 (14.5)	2196 (15.4)	523 (14.2)	141 (10.0)	104 (9.2)	
45-59y	1989 (9.7)	1344 (9.4)	340 (9.2)	153 (10.8)	152 (13.4)	
>60y	2597 (12.7)	1670 (11.7)	474 (12.8)	268 (19.0)	185 (16.3)	
Gender						
Female, n (%) ^b^	10,847 (53.6)	7599 (54.1)	1937 (52.9)	761 (54.2)	550 (48.7)	0.005
Place of residency						
Urban	16,357 (80.1)	11,147 (78.5)	3101 (84.2)	1164 (82.6)	945 (83.5)	< 0.001
Rural	4053 (19.8)	3, 044 (21.4)	579 (15.7)	244 (17.3)	186 (16.4)	
SISBEN level ^c^						
1	17,641 (86.4)	12,277 (86.5)	3195 (86.8)	1192 (84.6)	977 (86.3)	0.028
2	1943 (9.5)	1352 (9.5)	317 (8.6)	167 (11.8)	107 (9.4)	
3	99 (0.5)	72 (0,5)	14 (0.3)	7 (0.5)	6 (0.5)	
Other	727 (3.5)	490 (3.4)	154 (4.1)	42 (2.9)	41 (3.6)	
Comorbidities						
Rhinitis	1209 (5.9)	709 (5.0)	248 (6.7)	132 (9.4)	120 (10.6)	< 0.001
Acute Bronchitis	350 (1.7)	187 (1.3)	74 (2.0)	52 (3.7)	37 (3.2)	< 0.001
COPD	2223 (10.9)	1081 (7.6)	475 (12.9)	340 (24.2)	327 (28.9)	< 0.001
Atopic Eczema	667 (3.2)	424 (2.9)	141 (3.8)	70 (4.9)	50 (4.4)	< 0.001
Pulmonary Emphysema	71 (0.3)	29 (0.2)	17 (0.4)	14 (1)	11 (1.9)	< 0.001

^a^ Pearson Chi-square test for proportions and Welch ANOVA test for continuous variables

^b^ There were 179 patients of which information about sex was not available

^c^ The System for Selecting Beneficiaries of Social Spending (SISBEN) is a proxy-means instrument for targeting social spending based in an assessment of the socio-economic vulnerability of families according to their living conditions. The first level involves the most deprived populations and the third the relatively less deprived

Costs were reported both as their arithmetic mean with standard deviation (SD) and median with interquartile range (IQR). The Shapiro-Wilk test was used to test for normal distribution of the cost data and the Levene test to evaluate homogeneity of variances. The International Society of Pharmacoeconomics and Outcomes Research – ISPOR good research practice guidelines were used for cost data analysis [42]. As the sample/arithmetic mean cost is considered to be the most appropriate and informative measure for health care policy-makers, the Welch analysis of variance- ANOVA test and the Games-Howell test for post hoc analyses were used for analyzing mean costs among disease severity categories despite unequal variances and a non-normal distribution were found in the cost data [43–45]. In addition, as recommended by Mihaylova et al. [46] and highlighted by Gray et al. [47], “simple methods” (assuming normal distributions for costs) should be preferred when sample sizes are sufficiently large for the central limit theorem to exert itself.

To identify factors influencing asthma-related costs in the study population, expenditures were also analyzed in a one-part generalized linear model (GLM) with gamma distribution, log-link function and robust standard errors. GLM models with gamma distribution are considered to be the most suitable option for cost data analysis due to the advantage of analyzing both the mean and variance functions on the original dollar scale and addressing the frequently right-skewed distribution of cost data [46–49]. The association of the different sociodemographic covariates (i.e., place of residency, co-morbidities, etc.) and disease severity with asthma-related costs (as dependent variable) was explored by univariate analysis. Potential confounders (age, gender and SES) and those predictors with a *p*-value < 0.1 were included in the multivariate model. Estimated coefficients were reported as cost ratios (exponentiated form) which can be interpreted as a ratio of adjusted costs between the category of interest versus the category of reference for binary predictors or as the percentage of increase in the mean cost per unit increase in a continuous covariate [50–52]. A p-value less than 0.05 was considered statistically significant. Predicted adjusted costs were also used for comparisons in regard to clinical and sociodemographic features.

## Results

### Sample characteristics

The identification and selection process of the study sample is shown in Fig. 1. A final sample of 20,410 patients was analyzed. Frequencies of disease severity categories were: 69.5%, 18.0%, 6.9% and 5.5% for mild intermittent, mild persistent, moderate persistent and severe persistent asthma, respectively. As shown in Table 1, distribution of socio-demographic features and co-morbidities was significantly different among asthma severity categories. Living in an urban setting and having a low socioeconomic status (patients in the first category of SISBEN index) were predominant characteristics among all severity stages. With regard to comorbidities, atopic eczema and COPD cases were concentrated in the 0-4 yr. (45.2%) and > 60 yr. (50.7%) age groups, respectively (see Additional file 2). The estimated prevalence for atopic eczema and COPD for these age groups was 4.1% and 43.4%, respectively.

### Health care resource utilization

General physician visit (57.2%) was the most commonly used medical service and emergency department (ED) visit, the least (3.5%). Frequency distribution of asthma-related services in regard to disease severity is shown in Table 2. The overall frequency of HCRU increased significantly with disease severity (p for a trend = 0.026), but it was only evident among persistent asthma categories. When analyzed individually, the use of most services also increased significantly with disease severity in contrast to ED visits that showed a negative trend.

**Table 2:** Health care services and medication utilization related to severity

	Total *n* = 20,410	Mild intermittent *n* = 14,191	Mild Persistent *n* = 3680	Moderate Persistent *n* = 1408	Severe persistent *n* = 1131	*p*-value^a^
Medical services (n, %)^b^						
ED visits*	721 (3.5)	–	462 (12.5)	163 (11.5)	96 (8.5)	< 0.001
Mean (SD)^c^	1.1 (0.5)	–	1 (0)	1.3 (0.5)	1.4 (1.1)	
Hospitalizations*	1137 (5.5)	–	495 (13.4)	278 (19.7)	364 (32.1)	< 0.001
Mean (SD)^c^	1.6 (1.6)	–	1 (0)	1.5 (0.6)	2.5 (2.6)	
Specialized physician visits	5764 (28.2)	3539 (24.9)	1057 (28.7)	578 (41.0)	590 (52.1)	< 0.001
General physician visits	11,685 (57.2)	8445 (59.5)	1761 (47.8)	755 (53.6)	724 (64.1)	< 0.001
Other ambulatory services	1653 (8.1)	1009 (7.1)	297 (8.0)	160 (11.3)	187 (16.5)	< 0.001
Total frequency of any medical service	17,059 (83.5)	11,933 (84.1)	2923 (79.4)	1167 (82.8)	1036 (91.6)	0.026
Asthma medication prescriptions (n, %)^b^						
Controller medications						
ICS	4841 (23.7)	1777 (12.5)	1410 (38.3)	821 (58.3)	833 (73.6)	< 0.001
ICS + LABA	487 (2.4)	32 (0.2)	88 (2.4)	171 (12.1)	196 (17.3)	< 0.001
LABA	188 (0.9)	18 (0.1)	65 (1.7)	38 (2.7)	67 (5.9)	< 0.001
LM	660 (3.2)	0 (0)	221 (6.0)	154 (10.9)	285 (25.2)	< 0.001
Theophylline	240 (1.1)	36 (0.2)	62 (1.6)	63 (4.4)	79 (6.9)	< 0.001
Rescue Medications						
Oral corticosteroids	4594 (22.5)	399 (2.8)	2236 (60.7)	998 (70.8)	961 (84.9)	< 0.001
SABA	4911 (24.0)	1242 (8.7)	1798 (48.8)	946 (67.2)	925 (81.8)	< 0.001
Total frequency of any medication	9509 (46.6)	3880 (27.3)	3183 (86.5)	1336 (94.9)	1110 (98.1)	< 0.001

*For ED visits and hospitalizations trend analysis was conducted for mild to severe persistent categories

^a^Cochran-Armitage test. P for trend is reported

^b^The number of patients using each medical service is reported. Relative frequencies were calculated using the total number of subjects (N) for each column as denominator. Patients may have used more than one service in the cost analysis period

^c^Mean number of times that a patient required to use this medical service during the cost analysis period

*ED* emergency department; *ICS* inhaled corticosteroids; *ICS + LABA* inhaled corticosteroids-long acting B2 agonist combination; *LABA* long acting B2 agonist; *LM* leukotriene modifiers; *SABA* short acting B2 agonist

### Unadjusted direct costs according to disease severity

Mean costs of asthma-related medical services and medications increased in regard to disease severity (Table 3) except for ED visits that showed no differences (*p* = 0.187). The estimated mean annual (SD) direct cost *per patient* was I$331 (1278). Costs for mild intermittent, mild persistent, moderate and severe persistent asthma were I$67 (134), I$482 (1506), I$1061 (SD 1983) and I$2235 (SD 3426) respectively (*p* < 0.001). Among medical services and medications, hospitalizations and SABA treatment had the highest mean costs, respectively. Mean and median direct costs among only those who used asthma-related resources are presented in Additional files 3 and 4, respectively. Significant differences were found among costs distributed by age groups (p < 0.001) and the highest costs were observed for patients older than 60 yr. (I$533) (1942), those between 45 and 59 yr. (I$391) (1160) and children ≤4 years old (I$352) (1233).

**Table 3:** Unadjusted direct mean annual asthma-related mean costs by category of service and severity

Service ^a^	Total n = 20,410	Mild intermittent n = 14,191	Mild Persistent n = 3680	Moderate Persistent n = 1408	Severe persistent n = 1131	*p*-value ^b^
Medical services ^c^						
ED visits	$6 (39.3)	–	$21 (57)	$26 (78)	$20 (87)	0.187
Hospitalizations	$105 (946)	–	$239 (1492)	$368 (1838)	$662 (2027)	0.000
Specialized physician visits	$20 (47)	$15 (35)	$23 (52)	$38 (66)	$58 (91)	0.000
General physician visits	$37 (61)	$30 (39)	$41 (59)	$54 (77)	$100 (158)	0.000
Other ambulatory services	$7 (68)	$5 (61)	$9 (85)	$10 (49)	$22 (99)	0.000
Any medical service ^d^	$178 (958)	$51 (76)	$335 (1489)	$498 (1840)	$865 (2050)	0.000
Asthma medication prescriptions ^c^						
Controller medications						
ICS	$20 (124)	$5 (52)	$28 (117)	$84 (269)	$101 (306)	0.000
ICS + LABA	$24 (211)	$0.7 (19)	$12 (115)	$147 (513)	$208 (606)	0.000
LABA	$0.5 (16)	$0 (2)	$0.7 (14)	$0.8 (11)	$5 (62)	0.000
LM	$12 (89)	$0 (0)	$10 (61)	$37 (143)	$137 (296)	0.000
Rescue Medications						
Oral corticosteroids	$14 (74)	$0.3 (7)	$15 (36)	$48 (100)	$138 (249)	0.000
SABA	$25 (127)	$3 (27)	$38 (114)	$98 (231)	$166 (375)	0.000
Any medication ^e^	$152 (780)	$16 (108)	$147 (320)	$563 (895)	$1370 (2755)	0.000
Total mean costs ^f^	$331 (1278)	$67 (134)	$482 (1506)	$1061 (1983)	$2235 (3426)	0.000

^a^ Mean values and their (SD) are reported and were calculated using the total number of subjects in each column as denominator

^b^ Welch analysis of variance-ANOVA test

c One patient may contribute to the costs of different medical services or prescriptions

d, e, f Mean values represent the sum of costs derived from all medical services d, medications e (or both f ) presented during the cost-analysis period and divided by the total number of subjects in each disease category.

*ED* emergency department; *ICS* inhaled corticosteroids; *ICS+LABA* inhaled corticosteroids-long acting B2 agonist combination; *LABA* long acting B2 agonist; *LM* leukotriene modifiers; *SABA* short acting B2 agonist

**Fig. 2:**
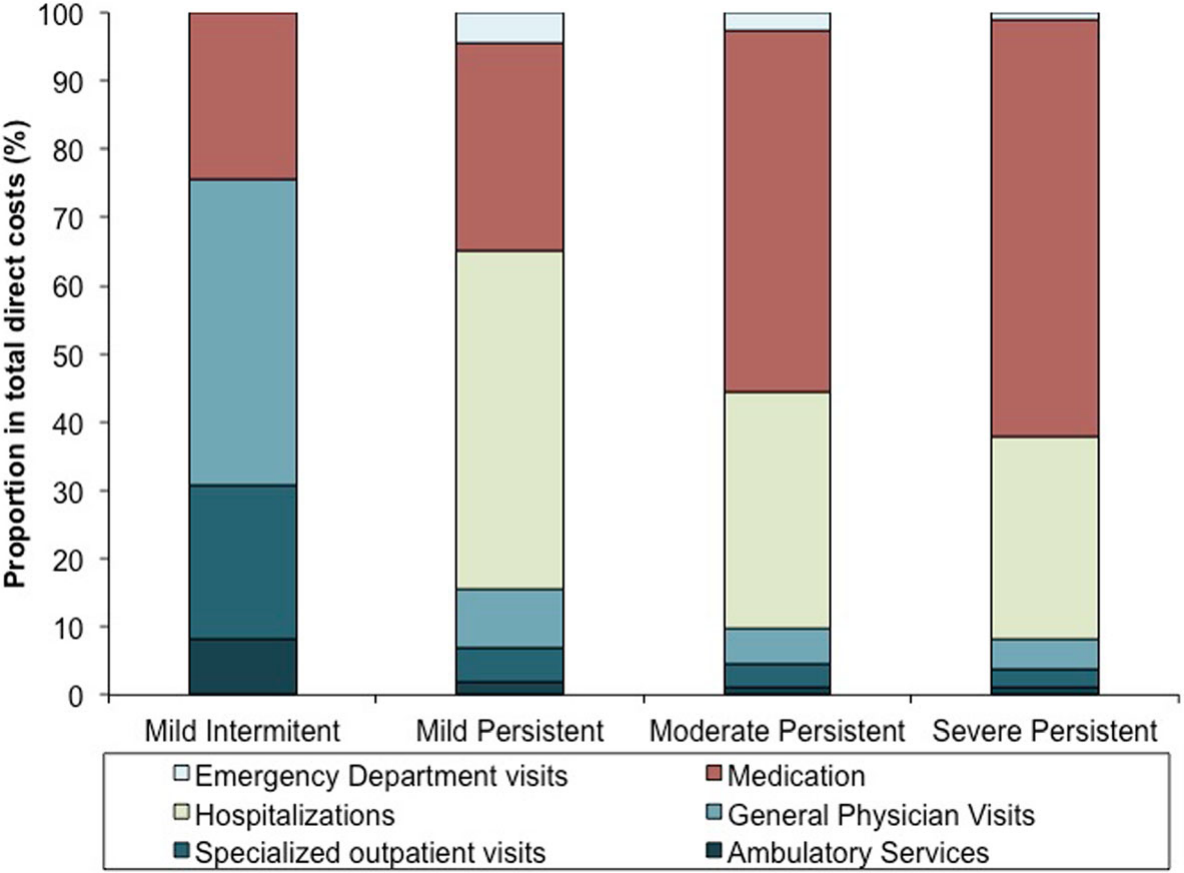
Distribution of total direct costs according to asthma severity. Cumulative percentage of total cost per category of severity among the different cost components

With respect to the distribution of costs according to the type of service and severity (Fig. 2), medication expenditures accounted for almost half of the total direct costs (46.1%) with the highest proportions in moderate and severe persistent groups (53% and 61.3%, respectively). Hospitalizations expenditures accounted for 31.8% of total costs and were more concentrated among patients in the mild persistent category (49.6%) compared to moderate and severe persistent (34.7% and 29.6%, respectively). The overall proportion of costs related to general physician visits was 11.4% and was higher among mild intermittent patients (44.7%), but decreased across the other three categories. Total annual direct costs were estimated to be I$6.7 million.

### Direct cost predictors

Having a comorbid condition (i.e., COPD, rhinitis, acute bronchitis), more severe asthma, belonging to highest-socioeconomic status (SISBEN 2–3 versus 1 as reference) and living in an urban setting were significant predictive factors for greater direct costs. No associations of costs with sex and age were found. Neither atopic eczema nor emphysema as co-morbidities were significantly related to direct costs (*p* = 0.066 and *p* = 0.064, respectively). The cost ratios (exponentiated form of estimated coefficients) of covariates are shown in Table 4. Adjusted mean (SD) cost of any patient with asthma was I$336 (615). Direct costs were higher in patients with severe persistent asthma compared to the other categories (*p* < 0.001). Adjusted mean costs stratified by different characteristics (age, gender, asthma severity, etc.) are shown in Table S5 (see Additional file 5).

**Table 4:** Generalized Linear Model (GLM) analysis of total direct costs (*N* = 19,509)

Parameter	Coefficient	Robust S.E.	*p*-value	95% CI	
Intercept	47.01	5.28	0.000	37.72	58.59
Age (continuous)	1.00	0.00	0.640	0.99	1.00
Female gender (ref: male gender)	0.97	0.02	0.372	0.92	1.03
Socioeconomic status (ref: Levels 2–3 in SISBEN index)					
Level 1	0.88	0.04	0.028	0.79	0.98
Rhinitis	1.76	0.12	0.000	1.53	2.02
Acute Bronchitis	1.34	0.15	0.009	1.07	1.67
Atopic eczema	1.12	0.06	0.066	0.99	1.26
COPD	1.65	0.10	0.000	1.46	1.87
Emphysema	1.29	0.18	0.064	0.98	1.71
Severity Index (ref: mild intermittent)					
Mild persistent asthma	6.63	0.35	0.000	5.97	7.36
Moderate Persistent asthma	13.59	0.77	0.000	12.15	15.20
Severe persistent	28.84	1.44	0.000	26.14	31.82
Urban residency (ref: rural residency)	1.15	0.43	0.000	1.07	1.24

*S.E.* standard error

### Sensitivity analysis

Three different sensitivity analyses were conducted. First, exclusion of asthmatic patients with any of the five selected comorbidities decreased total costs by 39.7% (from I$6.7 million to I$4 million). Asthmatic patients with COPD added I$1.9 million (or 29%) to direct costs of the total sample of patients with asthma. Analysis in the sub-groups indicated that the mean annual cost per patient decreased from I$391 to I$236 (39.6%) and I$533 to I$308 (42.2%) in the age groups of 45-59 yr. and > 60 yr., respectively. In addition, asthmatic patients with acute bronchitis added I$361,000 to total costs estimations and the exclusion of children with this co-morbidity decreased the mean cost by less than 2% (from I$352 to I$347) among those in the 0-4 yr. of age group.

## Discussion

This is the first claim-based study in Colombia that estimated the economic burden of asthma in terms of direct costs and its differences between disease severity categories. As expected, most severe cases of asthma incurred greater direct costs. Mean costs had a positive trend in relation with disease severity. Medication and hospitalizations accounted for approximately 78% of the total costs in the general cohort. In spite that patients with severe persistent asthma accounted for about 5% of the total sample, these cases contributed to more than a third of total costs (37%). Unadjusted as well as adjusted mean costs showed a positive trend with disease severity. Also, patients between 0 to 4 yrs. and > 60 yr. of age were significantly more likely to have had history of comorbidities and its effect in costs was considerable.

The influence of asthma severity and control levels in direct costs have been previously documented in some countries of Latin America [28, 53], but no information about Colombia has been published yet. The availability of large scale statistics of HCRU is mainly restricted to high-income countries [12, 54] and data are lacking for most low-and middle-income countries [2]. Gold et al. [55] used the Latin America Asthma Insights and Management Survey (LA AIM) to assess different levels of asthma control based on Global Initiative for Asthma (GINA) guidelines in five countries, and found that a poor control was a significant factor influencing health care resources use and greater medical costs, ranging from $70 for a well-controlled patient in Brazil, for an $5400 uncontrolled patient in Argentina (2013 US dollars). However, Colombia was not included in this study and therefore it is difficult to make a comparison of our results with previous estimations.

In addition, the Asthma Insights and Reality in Latin America (AIRLA) survey, conducted in 11 countries of the region, including Colombia, demonstrated the considerable lag in asthma care and control existing in these countries [28]. Based on the AIRLA survey, Neffen et al. [53], found that approximately 73.2% of annual costs of asthma-related health care for 10 countries in Latin America were due to unscheduled health care and overall related expenditure was higher among adults and children with severe persistent asthma symptoms (2010 US$558 and $769, respectively). Nevertheless, researchers were unable to include medication costs in this study; thus, estimations may underestimate the total economic impact in terms of direct costs. Even though these studies have contributed to the knowledge of asthma costs in the region, there is a potential limitation linked to their design (survey-based); frequencies may be underestimated, mainly because outcomes are reported by patients (recall bias) [56].

In Colombia, Hinestrosa et al. [57] conducted a retrospective analysis of clinical data (*n* = 2007) obtained from a hospital in a municipality of Colombia and found that between 2007 and 2009, medication was the main source of asthma-related direct cost (74%) derived mainly by inhaled corticosteroids. Similar results have been observed in previous studies in different countries, medication and hospitalization being the largest contributors. In 1985, hospital inpatient costs were estimated as the largest component cost of direct medical expenditures in the United States (44.6%) but, in 1994, medications had the higher proportions (40.1%) [58, 59]. Our study suggests a similar cost distribution with medications as the most important cost-deriver and SABA treatment accounting for the highest proportion of total cost among mild and moderate persistent asthma patients.

Living in an urban setting and higher SES were predictive factors for greater asthma expenditures in our study. Although there are many possible explanations (differences in the availability/proximity of health care services, level of education, environmental factors and even reverse causality) we were unable to identify causal relationships by means of this retrospective and cross-sectional study design [60]. With regard to comorbidities, the asthma and COPD overlap considerably increased costs compared to patients who had only an asthma diagnosis, indicating that COPD is also an important source of economic burden and this group of patients should be a target for policies aimed at reducing the burden associated with chronic respiratory diseases [61, 62].

The high prevalence of SABA treatment use in our findings may indicate poor adherence to controller medications or potential lack of knowledge about the efficacy of novel therapies in asthma treatment as well as established guidelines for asthma management by physicians in a clinical setting [63, 64]. The use frequency of SABA was similar to that of IC, but considerably high compared to IC + LABA combination. Together with a high rate of rescue medication, it was observed that IC prescriptions – the gold standard controller medication for persistent asthma [15] – were lower than expected. This improper management could also be explained by a low frequency of specialized physician visits even in those categorized as severe asthma (52%). In this regard, this scenario of poor asthma control and insufficient medical attention associated with elevated costs due to exacerbations and medication could imply the need for changes in Colombian policies for asthma treatment, including the creation of care management programs as observed in other Latin American countries [65].

This study has limitations in its methodology. Due to its focus on the third payer perspective, this work has restrictions to estimate epidemiologically important variables that may reflect asthma control. Frequency of exacerbations in asthmatic patients may be underestimated since many cases may have been treated at home. This could also explain why frequency of exacerbations are lower than those reported in the literature [30]. A negative trend in ED visits as disease severity increased may be explained by the positive direction in hospitalization frequencies. Our sample was not representative of the total population with asthma in Colombia; since our data was provided by an insurance company that offers health care services to less affluent inhabitants, estimations may be biased to reflect expenditures of this part of the population. Since indirect and direct non-medical asthma-related costs were not included, the total economic burden of asthma in Colombia is underestimated. Due to the nature of administrative databases, it is not possible in this type of study to assess disease control, nor to classify asthma severity according to the clinical criteria defined by internationally well-accepted guidelines or methods (i.e. GINA/ NAEPP guidelines or ERS/ATS) since claims information does not include direct clinical data inputs which usually are derived from medical charts, such as measures of daily symptoms, lung function, forced expiratory volume or peak expiratory flow [15, 18, 33]. However, the use of administrative claims data provides valuable information to estimate the magnitude in which asthma imposes an economic burden and it is, therefore, an important input for conducting economic evaluations [66, 67].

Although there is not a best-practice established algorithm for assessing disease severity in patients with asthma using administrative data, we considered that from those available in the literature, Leidy criteria is a reliable method for disease classification and the most suitable for analyzing information obtained from Colombian health system records. However, we aimed to optimize this classification adding exacerbation data, which are highly representative of disease severity. The implementation of an algorithm based in Leidy criteria in the Colombian context was possible due to the improvement of health care information systems and the availability of codified and documented data of health care utilization by patients. Limitations of prescription data for disease severity definition have been discussed by others [33]; for example, it does not ensure medication use by patients. In addition, prescriptions may be subjectively determined by the physician opinion and not accurately reflect asthma severity.

In the absence of empirical and clinical studies, this methodology can be further applied to other disease groups in the context of the developing world as a useful mechanism for generating evidence to health policy creation and evaluation. However, considerable improvements are required as there has been relatively little progress in data analysis and application despite a rapid rise of data production in this particular setting [68].

## Conclusions

Asthma severity is an important factor for increasing disease-related HCRU and direct costs. Our results demonstrate the considerable economic burden of asthma to health systems in the context of the developing world. Reinforcement of asthma control programs, with a focus on disease severity and the integral management of co-morbidities, may optimize the efficacy of intervention strategies and reduce costs to the health system.

## Additional files

Additional file 1: **Table S1.** Asthma medication (DOCX 18 kb)

Additional file 2: **Table S2.** Distribution of comorbidities according to age (DOCX 13 kb)

Additional file 3: **Table S3.** Unadjusted direct mean annual asthma-related costs among health care resource users (DOCX 20 kb)

Additional file 4: **Table S4.** Unadjusted direct median annual asthma-related costs among health care resource users (DOCX 20 kb)

Additional file 5: **Table S5.** Adjusted direct mean annual asthma-related costs by patient characteristics (DOCX 15 kb)

## Abbreviations

COPD: Chronic obstructive pulmonary disease
ED: Emergency department visit
GLM: Generalized Linear Model
HCRU: Health Care Resource Utilization
ICS + LABA: Inhaled corticosteroids-long acting B2 agonist combination
ICS: Inhaled corticosteroids
LABA: Long acting B2 agonist
OCS: Oral corticosteroid
PPA: Purchasing Power Parities
SABA: Short-acting β_2_-agonist
SISBEN: System for Selecting Beneficiaries of Social Spending
