# Supplementary material for: Health care costs and resource utilization for different asthma severity stages in Colombia: a claims data analysis
Source: World Allergy Organ J. 2018 Nov 12;11(1):26. doi: 10.1186/s40413-018-0205-4 (PMC6231276; doi:10.1186/s40413-018-0205-4)
Supplement: Supplementary file 1 — Table S1. Asthma medication (DOCX 18 kb) [file 40413_2018_205_MOESM1_ESM.docx]

**Supplementary Table 1.** Asthma medication

| **Controller medications** | **Inhaled corticosteroids** |
| --- | --- |
|  | Beclometasone |
|  | Budesonide |
|  | Ciclesonide |
|  | Dexamethasone |
|  | Fluticasone |
|  | Mometasone |
|  | **Inhaled corticosteroids /Long acting beta-agonists combination** |
|  | Fluticasone/Salmeterol |
|  | Budesonide/Formoterol |
|  | Mometasone/Formoterol |
|  | **Leukotriene Modifiers** |
|  | Montelukast |
|  | **Theophylliine** |
|  | **Long-acting beta-agonists** |
|  | Formoterol |
|  | Indacaterol |
|  | Salmeterol |
|  | **Omalizumab** |
| **Rescue medications** | **Oral/systemic corticosteroids** |
|  | Betamethasone |
|  | Dexametasone |
|  | Hydrocortisone |
|  | Methylprednisolone |
|  | Prendisolone |
|  | Prednisone |
|  | **Short acting beta-agonists** |
|  | Epinephrine |
|  | Levalbuterol |
|  | Terbutaline |
|  | Salbutamol |
