# Supplementary material for: Health care costs and resource utilization for different asthma severity stages in Colombia: a claims data analysis
Source: World Allergy Organ J. 2018 Nov 12;11(1):26. doi: 10.1186/s40413-018-0205-4 (PMC6231276; doi:10.1186/s40413-018-0205-4)
Supplement: Supplementary file 2 — Table S2. Distribution of comorbidities according to age (DOCX 13 kb) [file 40413_2018_205_MOESM2_ESM.docx]

|  | Rhinitis  (J30) | Acute Bronchitis  (J20) | COPD  (J44) | Atopic Eczema  (L20, L23) | Emphysema  (J43) |
| --- | --- | --- | --- | --- | --- |
| **N** | 1,209 | 350 | 2,223 | 667 | 71 |
| **Age group** | n (%)^a^ | | | | |
| 0-4y | 424 (35.1) | 96 (27.4) | 202 (9.1) | 302 (45.2) | 8 (11.2) |
| 5-9y | 346 (28.6) | 46 (13.1) | 135 (6.1) | 141 (21.1) | 11 (15.4) |
| 10-14y | 159 (13.1) | 20 (5.7) | 69 (3.1) | 44 (6.6) | 5 (7.0) |
| 15-19y | 70 (5.8) | 4 (1.1) | 37 (1.6) | 10 (1.5) | 3 (4.2) |
| 20-44y | 87 (7.2) | 25 (7.1) | 224 (10.1) | 33 (4.9) | 7 (9.8) |
| 45-59y | 74 (6.1) | 34 (9.7) | 428 (19.2) | 52 (7.8) | 13 (18.3) |
| >60y | 49 (4.0) | 125 (35.7) | 1,128 (50.7) | 85 (12.7) | 24 (33.8) |

**Supplementary Table 2.** Distribution of comorbidities according to age

^a^ The number of events in each age category and its respective frequency rate in parenthesis are shown. Relative frequencies were calculated as the proportion between cases and the total number of the respective co-morbidity (N).
