# Supplementary material for: Health care costs and resource utilization for different asthma severity stages in Colombia: a claims data analysis
Source: World Allergy Organ J. 2018 Nov 12;11(1):26. doi: 10.1186/s40413-018-0205-4 (PMC6231276; doi:10.1186/s40413-018-0205-4)
Supplement: Supplementary file 3 — Table S3. Unadjusted direct mean annual asthma-related costs among health care resource users (DOCX 20 kb) [file 40413_2018_205_MOESM3_ESM.docx]

| **Service** ^a^ | Total | Mild intermittent | Mild  Persistent | Moderate Persistent | Severe persistent | *p*-value ^b^ |
| --- | --- | --- | --- | --- | --- | --- |
| **Medical services** ^c^ |  |  |  |  |  |  |
| ED visits | $194 (86) | -- | $172 (13) | $228 (88) | $243 (188) | <0.001 |
| Hospitalizations | $1,889 (3,565) | -- | $1,778 (3,721) | $1,867 (3,789) | $2,057 (3,149) | 0.694 |
| Specialized physician visits | $73 (63) | $61 (47) | $82 (68) | $92 (75) | $112 (99) | <0.001 |
| General physician visits | $66 (68) | $50 (39) | $87 (58) | $101 (80) | $157 (174) | <0.001 |
| Other ambulatory services | $92 (223) | $78 (218) | $112 (281) | $94 (118) | $138 (210) | 0.002 |
| Any medical service ^d^ | $213 (1,045) | $60 (80) | $422 (1,660) | $601 (2,006) | $944 (2,124) | <0.001 |
| **Asthma medication prescriptions**  ^c^ |  |  |  |  |  |  |
| Controller medications |  |  |  |  |  |  |
| ICS | $85 (244) | $42 (143) | $73 (181) | $145 (340) | $137 (349) | <0.001 |
| ICS+LABA | $1,030 (913) | $322 (249) | $540 (526) | $1,215 (938) | $1,204 (963) | <0.001 |
| LABA | $58 (161) | $36 (66) | $40 (98) | $29 (65) | $97 (241) | 0.206 |
| LM | $371 (338) | $ (0) | $169 (192) | $342 (290) | $543 (359) | <0.001 |
| Rescue Medications |  |  |  |  |  |  |
| Oral corticosteroids | $62 (146) | $10 (44) | $26 (43) | $68 (112) | $163 (263) | <0.001 |
| SABA | $105 (243) | $39 (85) | $79 (153) | $146 (270) | $203 (406) | <0.001 |
| Any medication ^e^ | $328 (1,118) | $60 (201) | $170 (339) | $593 (909) | $1,396 (2,755) | <0.001 |
| **Total costs** ^f^ | $331 (1,278) | $67 (134) | $ 482 (1,506) | $1,061 (1,983) | $2,235 (3,426) | <0.001 |

**Supplementary Table 3.** Unadjusted direct mean annual asthma-related costs among health care resource users

^a^ Mean values and their (SD) are reported. Mean costs were calculated using the number of subjects who had service utilization or received a medication prescription as denominator.

^b^ Welch analysis of variance-ANOVA test

^c^ Patients may have costs resulting by the use of more than one service in the cost analysis period

^d, e, f^ Mean values represent the sum of costs derived from all medical services ^d^, medications ^e^ (or both ^f^ ) presented during the cost-analysis period and divided by the number of subjects in each disease category who used health care resources.

ED=emergency department; ICS=inhaled corticosteroids; ICS+LABA=inhaled corticosteroids-long acting B2 agonist combination; LABA=long acting B2 agonist; LM=leukotriene modifiers; SABA=short acting B2 agonist
