# Supplementary material for: Health care costs and resource utilization for different asthma severity stages in Colombia: a claims data analysis
Source: World Allergy Organ J. 2018 Nov 12;11(1):26. doi: 10.1186/s40413-018-0205-4 (PMC6231276; doi:10.1186/s40413-018-0205-4)
Supplement: Supplementary file 4 — Table S4. Unadjusted direct median annual asthma-related costs among health care resource users (DOCX 20 kb) [file 40413_2018_205_MOESM4_ESM.docx]

**Supplementary Table 4.** Unadjusted direct median annual asthma-related costs among health care resource users

| **Service ^a^** | Total | Mild  intermittent | Mild  Persistent | Moderate Persistent | Severe  persistent |
| --- | --- | --- | --- | --- | --- |
| **Medical services ^b^** |  |  |  |  |  |
| ED visits | $165 (165-202) | -- | $165 (165-166) | $166 (165-330) | $166 (165-202) |
| Hospitalizations | $904 (311-1,790) | -- | $782 (305-1,565) | $782 (260-1,590) | $1,043 (472-2,020) |
| Specialized physician visits | $59 (29-96) | $33 (29-87) | $62 (33-115) | $66 (33-120) | $91 (34-146) |
| General physician visits | $35 (35-79) | $35 (21-70) | $79 (35-114) | $79 (35-127) | $113 (56-193) |
| Ambulatory services | $38 (30-99) | $38 (30-76) | $38 (30-99) | $49 (31-107) | $76 (38-162) |
| Any medical service ^c^ | $35 (21-112) | $35 (21-70) | $114 (30-194) | $123 (35-297) | $248 (114-695) |
| **Asthma medication prescriptions ^b^** |  |  |  |  |  |
| Controller medications |  |  |  |  |  |
| ICS | $18 (3-55) | $11 (2-18) | $18 (5-50) | $36 (18-97) | $36 (18-97) |
| ICS+LABA | $789 (315-1,420) | $176 (157-493) | $369 (173-628) | $1,000 (530-1,717) | $1,061 (473-1,705) |
| LABA | $6 (6-19) | $6 (6-19) | $6 (6-19) | $13 (6-19) | $6 (6-25) |
| LM | $280 (140-551) | $0 (0) | $111 (70-210) | $217 (140-420) | $490 (280-770) |
| Rescue Medications |  |  |  |  |  |
| Oral corticosteroids | $22 (7-60) | $3 (1-7) | $15 (7-22) | $35 (16-73) | $98 (58-183) |
| SABA | $23 (12-59) | $12 (12-12) | $23 (12-47) | $35 (12-107) | $47 (23-131) |
| Any medication  ^d^ | $44 (12-200) | $12 (2-38) | $52 (17-140) | $205 (74-739) | $520 (194-1,670) |
| **Total median costs**  ^e^ | $62 (30-167) | $35 (22-79) | $181 (104-373) | $475 (231-1,251) | $1,168 (495-2,629) |

^a^ Median values and their (IQR) are reported. Median costs were calculated using the number of subjects who had service utilization or received a medication prescription as denominator.

^b^ Patients may have costs resulting by the use of more than one service in the cost analysis period

^c, d, e^ Mean values represent the sum of costs derived from all medical services ^c^, medications ^d^ (or both ^e^ ) presented during the cost-analysis period and divided by the number of subjects in each disease category who used health care resources.

ED=emergency department; ICS=inhaled corticosteroids; ICS+LABA=inhaled corticosteroids-long acting B2 agonist combination; LABA=long acting B2 agonist; LM=leukotriene modifiers; SABA=short acting B2 agonist
