# Supplementary material for: Health care costs and resource utilization for different asthma severity stages in Colombia: a claims data analysis
Source: World Allergy Organ J. 2018 Nov 12;11(1):26. doi: 10.1186/s40413-018-0205-4 (PMC6231276; doi:10.1186/s40413-018-0205-4)
Supplement: Supplementary file 5 — Table S5. Adjusted direct mean annual asthma-related costs by patient characteristics (DOCX 15 kb) [file 40413_2018_205_MOESM5_ESM.docx]

**Supplementary Table 5.** Adjusted direct mean annual asthma-related costs by patient characteristics

| **Parameter** | **Mean (SD)** |
| --- | --- |
| **Age groups** |  |
| 0-4y | 328 (538) |
| 5-9y | 281 (582) |
| 10-14y | 272 (524) |
| 15-19y | 247 (543) |
| 20-44y | 257 (517) |
| 45-59y | 426 (775) |
| >60y | 524 (833) |
| **Gender** |  |
| Female | 324 (596) |
| Male | 349 (635) |
| **Socioeconomic status** |  |
| SISBEN 1 | 325 (591) |
| SISBEN 2 – 3 | 427 (783) |
| **Severity categories** |  |
| Mild intermittent | 67 (18) |
| Mild persistent | 470 (149) |
| Moderate persistent | 1,064 (390) |
| Severe persistent | 2,346 (925) |
| **Urban vs rural** |  |
| Urban | 354 (637) |
| Rural | 260 (508) |
